# Predictive role of hematological indices in patients with acute coronary syndrome in Ethiopia: Intrahospital outcomes

**DOI:** 10.1016/j.heliyon.2024.e36790

**Published:** 2024-08-24

**Authors:** Samuel Tadesse, Elsah Tegene, Daniel Yilma, Tilahun Yemane, Esayas Kebede Gudina, Andualem Mossie

**Affiliations:** a Department of Biomedical Sciences, Jimma University, Ethiopia; b Department of Internal Medicine, Jimma University, Ethiopia; c Department of Medical Laboratory, Jimma University, Ethiopia

**Keywords:** Risk of mortality, Prognosis, Haematological parameters, Acute coronary syndrome

## Abstract

**Background:**

Apart from troponins, various additional biomarkers that indicate myocardial injury, inflammation, thrombosis, and other routes are being studied to improve the treatment of acute coronary syndrome (ACS). Myeloid activity has been found to be elevated in ACS, and this has sparked a great deal of interest in hematological parameters since they might offer independent insights into pathophysiology and risk assessment.

**Objective:**

The purpose of this study was to evaluate the hematological markers' prognostic ability for all intrahospital causes of mortality in individuals with an ACS diagnosis.

**Methods:**

A long-term cohort study based at an institution was done. At Jimma Medical Center, patients with an ACS diagnosis were progressively brought in between May 1, 2022, and October 31, 2023. Complete blood counts (CBC) and biochemical analysis were carried out. Multilevel mixed effect logistic regression was computed to evaluate the predictive competence of hematological indices on intrahospital mortality. Prognostic performance of hematological parameters was done using the ROC curve analysis.

**Result:**

A total of 110 patients were included, of which 99 (90 %) were diagnosed ST-elevation myocardial infarction, and 74 (67.3 %) were men. The mean age was 56 (±11) years. RDW, platelet count, and MCV were independently associated with intrahospital mortality (AOR = 1.20 with P < 0.001, AOR = 0.995 with P < 0.03, and AOR = 0.897 with P < 0.025, respectively). The predictive power of RDW-SD for intrahospital mortality was evaluated by ROC analysis, the AUC value were 0.737 (95 % CI 0.669–0.805).

**Conclusion:**

This study found that red cell distribution width, mean corpuscular volume, and platelets were predictive factors for intrahospital death in patients with ACS. Thus, it is possible to predict the prognosis of an ACS patient using hematological data.

## 1 Introduction

Acute coronary syndrome, primarily resulting from atherosclerosis, is among the main reasons of death globally and typically requires prompt medical attention [1]. Although the pathogenesis of ACS varies greatly, most instances are related to the rupture of an atherosclerotic plaque and total or partial blockage of the artery connected to the infarct. High-sensitivity troponin-I, which has a greater sensitivity and negative predictive value, has fundamentally altered the diagnosis of ACS. However, problems with consistency, meaning, and precision still remain. To enhance the treatment of acute coronary syndrome, a variety of biomarkers for inflammation, thrombosis, myocardial damage, and other pathways are being studied in addition to troponins [2].

Thus, prognostic instruments and risk stratification for patients with ACS can be established using inflammatory biomarkers associated with leukocytes and platelets [3]. Increased myeloid cell activity associated with ACS was caused by inflammatory processes, which raised erythropoietin levels. Low levels of chronic inflammation lead to plaque instability and thrombus formation, which play a significant role in the initiation and development of atherosclerotic plaques [4]. Myeloid activity has been found to be elevated in ACS, and this has sparked a great deal of interest in hematological parameters since they might offer independent insights into pathophysiology and risk assessment [5].

In a typical complete blood count, red blood cell distribution width (RDW), a measurement of red blood cell volume fluctuations (anisocytosis), is provided. Recent research has demonstrated the importance of RDW as a predictor of worse than ideal therapeutic outcomes in the setting of several illnesses, including ACS [6]. Among patients admitted with ACS, elevated RDW is a reliable indicator of death and significant adverse cardiac events [7]. A growing body of evidence was describing the relationship between RDW and prognosis in patients with stable coronary artery disease (CAD) and myocardial infarction [8,9]. The middling size of erythrocytes is measured by mean corpuscular volume (MCV), which is directly correlated with erythrocyte diseases. Research indicates that the prognosis of multiple disorders, such as cerebral ischemic stroke, coronary artery disease (CAD), and peripheral artery disease, is correlated with MCV [10].

A greater mean platelet volume (MPV), a measure of platelet activity, has been shown to be necessary in order to detect a cardiovascular event. Elevated MPV can predict the risk of acute myocardial infarction (AMI), mortality after MI, and re-stenosis after coronary angioplasty, as Chu SG et al. have shown [11]. In addition to MPV, leukocyte count, neutrophil to lymphocyte ratio (NLR), platelet count, platelet to lymphocyte ratio (PLR), and white blood cell (WBC) to MPV ratio (WMR) are other potentially helpful complete blood count indicators [12]. The primary focus of earlier research has been on how these biomarkers relate to the long-term mortality and problems that ACS patients may experience. Therefore, the purpose of this prospective research was to test if hematological markers could be useful in forecasting the intrahospital mortality of patients who have been diagnosed with ACS.

## 2 Materials and methods

### 2.1 Research design and data collection

This study was conducted in the cardiovascular unit of the Jimma Medical Center (JMC) from May 1, 2022, to October 31, 2023, a period of eighteen months. In southwest Ethiopia, JMC is one of the largest and most reputable teaching and referral hospitals. To ascertain the prognostic significance of hematological markers in patients with ACS, prospective longitudinal cohort research with an institutional basis was conducted. Included were consecutive individuals who were admitted during the study period and had confirmed cases of ACS. Patients with pre-existing medical conditions such as hematological disorders including blood cancer, autoimmune or systemic inflammatory diseases, chronic liver disease, immunosuppressive and anticoagulant medication users, patients under the age of eighteen, and readmissions following discharge were excluded [13].

Within 24 h of an ACS patient's admission, baseline data were collected, and the patient was checked for compliance with all inclusion and exclusion criteria. After then, patients were monitored to determine the importance of hematologic markers for prognosis until they were discharged or passed away. In-person interviews were utilized to gather demographic and health-related data using a standardized questionnaire that had been meticulously adapted and taken from other studies [4,14].

#### 2.1.1 Laboratory analysis

Four milliliters venous blood sample was taken in potentially aseptic circumstances upon admission. Qualified laboratory technicians performed complete blood counts (CBC) and serum biochemistry analyses on 2 mL of blood each. The Uni-CelDxH 800 Coulter Cellular Analysis System was used to assess whole blood counts, which included hemoglobin, RBC, RDW, WBC, and WBC differential counts (neutrophil, lymphocyte, eosinophil, basophil, and monocyte), Platelet, and MPV. Calculations were made for NLR, PLR, MPVLR, and WBC to MPV (WMR). Relevant serum biochemical markers were found using a Roche Cobas Integra-400 processor, including high-sensitive troponin I and serum creatinine. Every 72 h from the time of admission until the time of discharge, a blood sample was taken in the morning. Within 30 min of the blood sample, all measurements were completed. The standard operating procedures were followed to guarantee the quality and analysis of the blood sample. Reports were reviewed every day for confidentiality and completeness for every laboratory inquiry.

#### 2.1.2 Statistical analysis

To analyze the data, Stata-SE version 14 was utilized. For continuous variables, the report format was mean ± SD. Both precise values and percentages were used to describe categorical variables. To confirm the assumption, a normality test was performed. To assess the mortality predictors of these factors, multilevel mixed effect univariate logistic regressions were carried out. The independent predictors of mortality were found using a multilevel mixed effect multivariate logistic regression model. To ascertain the predictive accuracy (specificity and sensitivity) of hematological markers in predicting short-term mortality, ROC curve analysis was performed. To determine a cut-off value for optimizing the sensitivity and specificity of variables, the Youden Index was also utilized. A cutoff of p < 0.05 was established as the accepted measure of statistical significance.

### 2.2 Ethical considerations

After examination, the study protocol was accepted by the Jimma University Institutional Research Board in accordance with the Declaration of Helsinki. It was then assigned the number IHRPGD/554/2022. The study participants (or, in the case that the patient is unable to communicate or give consent, their family member or caregiver) were informed of the purpose of the study and the importance of their cooperation. Each participant provided signed, informed consent prior to any data collection.

## 3 Result

### 3.1 Socio-demographic and clinical characteristics of ACS patients

Of the 110 ACS patients included in this study, 74 (67.27 %) were male and the average age was 56.69 ± 11.91 years. Most of the patients 99 (90 %) had STEMI diagnoses and 60 (54.5 %) were urban residents. Systemic arterial hypertension, khat chewing, and diabetes mellitus were the three most prevalent risk factors of ACS patients. Aspirin 108 (98.2 %), first anticoagulants therapy given following hospitalization 103 (93.6 %), statin 95 (86.4 %), and P_2_Y_12_ receptor antagonist 88 (80.0 %) were the most often given pharmaceutical medications (Table 1). The study participants' demographic and health-related details were covered in a previously published article [13].

**Table 1:** The research participants' demographic information and health-related attributes.

Characteristics	Statistics
Age (mean ± SD)	56.69 ± 11.91
Gender: Male	74 (67.27 %)
Residence: Urban	60 (54.55 %)
Occupation: Farmer	47 (42.7 %)
Body mass index (kg/m^2^) (mean ± SD)	24.0 ± 3.5
WHR (mean ± SD)	1.0 ± 0.09
STEMI	99 (90 %)
NSTEMI	9 (8.2 %)
Unstable Angina	2 (1.8 %)
Risk factors	
Hypertension	50 (45.5 %)
Diabetes mellitus	33 (30.0 %)
Kidney disease	2 (1.8 %)
Family history of coronary artery disease	13 (11.8 %)
Khat chewing	50 (45.5 %)
Alcohol	18 (16.36 %)
Smoking	20 (18.2 %)
Sedentary lifestyle	25 (22.7 %)
Killip score	
Class I	39 (35.4 %)
Class II	32 (29.1 %)
Class III	21 (19.1 %)
Class IV	18 (16.4 %)
HEART score	
Low risk	7 (6.4 %)
Intermediate risk	43 (39.1)
High risk	60 (54.5 %)
GRACE score	
Low risk	28 (25.5 %)
Intermediate risk	45 (40.9 %)
High risk	37 (33.6 %)
Treatment following hospitalization	
Aspirin	108 (98.2 %)
Anticoagulants therapy	103 (93.6 %)
Statin	95 (86.4 %)
P2Y12 receptor antagonist	88 (80.0 %)
ACE inhibitors	65 (59.1 %)
Morphine	59 (53.6 %)
Oxygen Supplement	57 (51.8 %)

WHR, waist hip ratio; STEMI, ST-elevation myocardial infarction.

Based on patient outcomes, comparisons were also made between health-related traits, haematological indices, and biochemical markers. While monthly income was considerably higher in the ACS survivor group, the non-survivor group's mean disparities in HEART and GRACE scores were much higher. There was no visible variation in the incidence of co-morbid disorders between ACS survivor and non-survivor group. The ACS non-survivor group had greater mean differences in RDW-SD, MPV, neutrophil count, WBC to MPV ratio (WMR), creatinine, and high sensitive troponin-I, while the ACS survivor group had higher mean differences in mean corpuscular volume (MCV) (Table 2).

**Table 2:** Comparison of health-related characteristics, haematological, and biochemical parameters among ACS survivors and non-survivor groups.

Variables	ACS	ACS
	Survivors *(Mean ±SD)*	Non-Survivors *(Mean ±SD)*
Age	56.5 ± 12.1	57.2 ± 10.5
BMI	24.0 ± 3.6	23.7 ± 3.1
WHR	1.0 ± 0.09	1.0 ± 0.08
HEART score^a^	6.2 ± 1.5	7.0 ± 1.6
GRACE score,^a^ ETB	123.3 ± 32.4	142.9 ± 31.5
Income per month^a^	5930.3 ± 5076.2	3990.0 ± 3558.9
WBC count *10*^*3*^*/μl*	9.9 ± 4.1	11.7 ± 4.7
RBC count *10*^*6*^*/μl*	4.6 ± 0.7	4.7 ± 0.7
Hemoglobin gm/dl	13.5 ± 2.2	13.3 ± 2.2
Hematocrit- %	40.5 ± 6.7	39.9 ± 6.8
MCV *fl*	86.9 ± 6.1	83.8 ± 11.5
MCH *pg*	28.9 ± 2.4	28.5 ± 2.0
MCHC *mg/dl*	33.2 ± 2.1	33.4 ± 1.8
RDW-SD *fl*	52.8 ± 8.3	60.0 ± 8.1
Platelet count *10*^*3*^*/μl*	272.7 ± 128.9	239.4 ± 103.5
MPV *fl*	10.5 ± 1.2	11.0 ± 1.3
Neutrophil count *10*^*3*^*/μl*	7.6 ± 3.7	9.6 ± 4.8
Lymphocyte count *10*^*3*^*/μl*	1.2 ± 0.8	1.2 ± 0.7
Monocyte count *10*^*3*^*/μl*	0.6 ± 0.7	0.7 ± 0.4
Eosinophil count *10*^*3*^*/μl*	0.1 ± 0.1	0.14 ± 0.18
Basophil count *10*^*3*^*/μl*	0.05 ± 0.1	0.05 ± 0.09
Neutrophil to lymphocyte ratio	10.4 ± 17.0	12.4 ± 14.3
Platelet to lymphocyte ratio	354.3 ± 620.4	272.0 ± 225.7
WBC to MPV ratio	0.9 ± 0.4	1.0 ± 0.4
MPV to lymphocyte ratio	13.4 ± 15.4	13.4 ± 13.5
High sensitive troponin-I, μg/dl	37.7 ± 39.6	120.5 ± 127.8
Creatinine, mg/dl	0.9 ± 0.4	1.8 ± 1.5

BMI, body mass index; WHR, waist hip ratio; MCV, Mean corpuscular volume; MCH, Mean Corpuscular Hemoglobin; MCHC, Mean corpuscular hemoglobin concentration; RDW, Red cell distribution width; MPV, Mean Platelet Volume. ETB, Ethiopian Birr.

a Significant mean difference (*t*-test).

### 3.2 Predictive role of hematological markers

Hematological and serum biochemical markers were assessed as mortality predictors using multilevel mixed effect univariate logistic regressions. To find viable candidate variables for the final model, the univariable analysis's p-value of less than 0.25 was consulted. Accordingly WBC, MCV, RDW-SD, platelet count, MPV, neutrophil count, WMR, creatinine, and high sensitive troponin-I were found as a predictor of intrahospital mortality in patients with ACS (p < 0.25) (Table 3). To evaluate independent predictors of death, we ran a multilevel mixed effect multivariate logistic regression model. Consequently, it was found that platelet count (AOR 0.995, 95 % CI 0.991–0.999, p < 0.030), MCV (AOR 0.897, 95 % CI 0.815–0.986, p < 0.001), and RDW-SD (AOR 1.2, 95 % CI 1.11–1.29, p < 0.001) were factors independently predicted short-term (intrahospital) mortality (Table 4).

**Table 3:** Multilevel mixed-effects univariate logistic regression to determine the predictors of intrahospital mortality.

Variables	Crude Odds Ratio	Std. Err.	95 % Conf. Interval	P- Value
WBCcount	1.092	0.034	1.028279–1.160502	0.004
RBC count	1.016	0.188	0.7073654–1.460207	0.930
Hemoglobin	0.975	0.061	0.8616622–1.102243	0.682
Hematocrit	0.988	0.021	0.9480546–1.029927	0.572
MCV	0.948	0.021	0.9086819–0.9891998	0.014
MCH	0.932	0.055	0.8304303–1.045551	0.229
MCHC	1.050	0.072	0.9182283–1.201074	0.475
RDW-SD	1.188	0.033	1.125066–1.254538	0.001
Platelet count	0.997	0.001	0.9946252–1.000138	0.063
MPV	1.315	0.144	1.06116–1.629696	0.012
Neutrophil count	1.119	0.038	1.048477–1.195681	0.001
Lymphocyte count	0.911	0.163	0.6424401–1.292547	0.602
Monocyte count	1.031	0.200	0.7046776–1.508476	0.875
Eosinophil count	1.758	1.308	0.4087543–7.563278	0.448
Basophil count	0.820	1.030	0.0699111–9.618038	0.874
Neutrophil to lymphocyte ratio	1.005	0.008	0.9913116–1.02076	0.429
Platelet to lymphocyte ratio	0.999	0.001	0.9984802–1.000513	0.331
WBC to MPV ratio	1.920	0.597	1.043496–3.533502	0.036
MPV to lymphocyte ratio	0.999	0.009	0.9812788–1.018607	0.981
Creatinine	2.694	0.543	1.815298–4.000891	0.001
High Sensitive Troponin -I	1.029	0.005	1.020635–1.039349	0.001

MCHC, Mean corpuscular hemoglobin concentration; RDW-SD, Red cell distribution width standard deviation.

**Table 4:** Multilevel mixed-effects multivariate logistic regression to determine the independent predictors of intrahospital mortality.

Variables	Adjusted Odds Ratio	Std. Err.	95 % Conf. Interval	P- Value
White blood cell count	1.083	0.442	0.4871263–2.410128	0.844
Mean corpuscular volume	0.897	0.044	0.8152799–0.9864688	0.025
Mean corpuscular hemoglobin	1.127	0.145	0.8760933–1.448852	0.353
RDW-SD	1.202	0.046	1.115206–1.296134	0.001
Platelet count	0.995	0.002	0.9912565–0.99955	0.030
Mean platelet volume	0.986	0.489	0.372004–2.611317	0.977
Neutrophil count	0.952	0.129	0.7290406–1.244001	0.720
WBC to MPV ratio	4.246	16.158	0.0024497–7360.939	0.704
Creatinine	2.601	0.838	1.383049–4.891159	0.003
High Sensitive Troponin -I	1.029	0.005	1.018908–1.03945	0.001

RDW-SD, Red cell distribution width standard deviation.

**Fig. 1:**
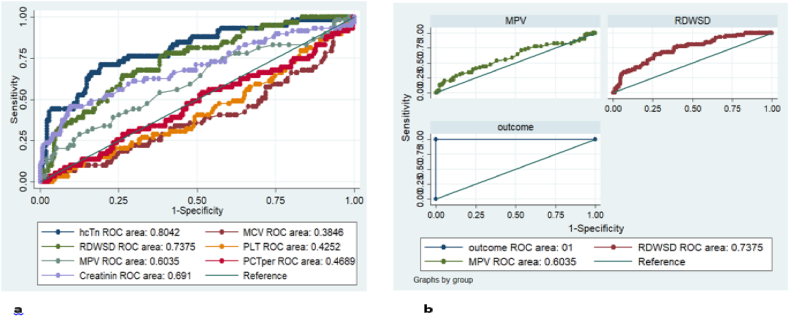
ROC curve output: (a) intrahospital mortality predicting power of the hematological and biochemical markers; and (b) intrahospital mortality predicting power of MPV and RDWSD.

**Fig. 2:**
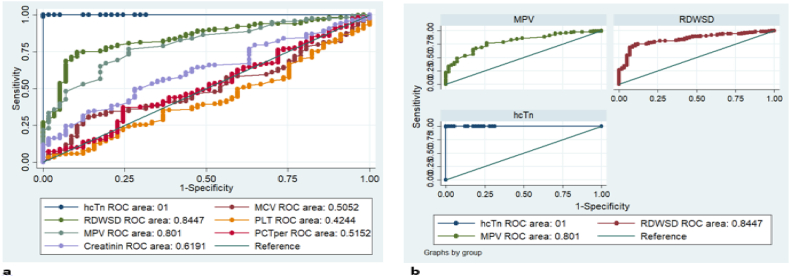
ROC curve output: (a) prognostic potential of biochemical and hematological markers in comparison to the highly sensitive troponin-I; and (b) prognostic potential of RDWSD and MPV in comparison to the highly sensitive troponin-I.

The predictive accuracy of hematological markers in short-term mortality prediction was assessed using ROC curve analysis. RDW-SD (AUC = 0.737, 95 % CI 0.669–0.805, p < 0.001) and MPV (AUC = 0.603, 95 % CI 0.518–0.688, p < 0.001) exhibited the strongest discriminative ability to predict short-term mortality. For RDW-SD and MPV, a cut-off value of >54.6 and > 10.7, respectively, was established in order to maximize the specificity and sensitivity (Table 5 and Fig. 1(a and b)). The gold standard high sensitive troponin-I was compared with those predictive variables using ROC-gold analysis. Accordingly, the most discriminative abilities as high sensitive troponin-I were exhibited by RDW-SD (AUC = 0.845, 95 % CI 0.794–0.894, p < 0.001) and MPV (AUC = 0.8014, 95 % CI 0.744–0.858, p < 0.001). (Table 6 and Fig. 2(a and b)).

**Table 5:** ROC analysis of predictive value of intrahospital mortality.

Variables	ROC Area	Std. Err	95 % Conf. Interval	Cut-off	Sensitivity (%)	Specificity (%)
Platelet count	0.4252	0.0416	0.34359–0.50682	212	50.00	35.19
MCV	0.3846	0.0424	0.30137–0.46774	85.7	40.00	40.00
MPV	0.6035	0.0433	0.51852–0.68845	10.7	60.00	52.22
Creatinine	0.6910	0.0435	0.60604–0.77588	0.91	70.00	50.37
RDW-SD	0.7375	0.0348	0.66935–0.80573	54.67	77.97	60.00
Hs-T-n	0.8042	0.0331	0.73935–0.86900	43.51	75.00	71.85

MCV, Mean corpuscular volume; MPV, Mean Platelet Volume; RDW-SD, Red cell distribution width standard deviation.

**Table 6:** ROC analysis of variables compared with the gold standard high sensitive troponin-I.

Variables	ROC Area	Std. Err	[95 % Conf. Interval]	chi2	P- Value
Platelet count	0.4244	0.0399	0.34621–0.50260	208.1438	0.001
MCV	0.5052	0.0378	0.43112–0.57920	171.6051	0.001
MPV	0.8014	0.0292	0.74417–0.85856	46.3381	0.001
Creatinine	0.6191	0.0375	0.54556–0.69258	103.1568	0.001
RDW-SD	0.8447	0.0254	0.79492–0.89438	37.4853	0.001
Hs-T-n (standard)	1.0000	0.0000	1.00000–1.00000		

MCV, Mean corpuscular volume; MPV, Mean Platelet Volume; RDW-SD, Red cell distribution width standard deviation.

## 4 Discussion

The level of hypoxemia and multifactorial-originated inflammation that ACS patients endure during their hospital stay is what causes the hematopoietic feedback. It is linked to increased myeloid activity, which in turn causes the bone marrow to release hematopoietic stem cells and aids in the activation of atherosclerotic plaques. The primary finding of this study was the significance of routine evaluations of platelet count, MCV, MPV, RDW, and RDW for the prognosis of intrahospital ACS patients. Depending on how the recommended treatment is accepted, these variables indicate the extent of systemic damage and, subsequently, the clinical outcome at any given time [5].

This is the first study of the relationship between ACS prognosis and hematological indicators in the study area. As compared to survivors, the ACS non-survivor group had significantly higher HEART and GRACE scores, according to this study. In line with this finding, a research carried out by Gutiérrezour VHC revealed a favorable association between the GRACE risk score and in-hospital mortality [15,16]. Studies examining hematological markers in relation to SYNTAX, GRACE, KILLP, and TIMI risk scores have discovered a relationship between these markers and intrahospital mortality in ACS patients [17,18].

The biomarker study results indicated that ACS non-survivor patients had a decreased MCV value (P = 0.0014), but significantly higher WBC count, RDW-SD, MPV, neutrophil count, and WMR (P = 0.004, 0.000, 0.012, 0.001, and 0.036, respectively). Numerous studies have shown that blood count indices, such as high NLR, high PLR, MPV, RDW, neutrophil count, and MPV, are effective at predicting cardiovascular morbidity and mortality [[19], [20], [21]]. According to a statistical model used in this investigation, the intrahospital mortality of patients with ACS was independently predicted by platelet count, MCV, and RDW. RDW is important as a predictor of worse than ideal clinical outcomes in the setting of several illnesses, including CAD, according to a number of recent research [8,22,23]. In a study by Cavusoglu et al., it was found that the RDW was a very reliable standalone predictor of all-cause death in ACS patients. According to Tonelli et al., mortality rates were considerably higher in patients with elevated RDW among CAD patients. The relationship between worse outcomes among cardiovascular illnesses and greater RDW values has been explained by a number of tenable hypotheses. The most fascinating of these are related to the ways in which elevated RDW influences vascular damage, endothelial dysfunction, and changes in the cholesterol content of the RBC membrane—all of which are implicated in the onset, advancement, and instability of atherosclerotic plaque [[24], [25], [26]].

One independent predictor of intra-hospital mortality identified in this investigation was a reduction in mean corpuscular volume. Unlike the current study, research on the long-term outcomes of ACS patients by Cheng L et al. indicated that a higher MCV at admission is a significant and reliable predictor of MACEs in ACS patients. Nevertheless, the admission MCV's capacity to forecast MACEs declined as the follow-up period increased [10]. Another Chinese investigation also demonstrated that longer-term MACEs were independently predicted by greater admission MCV and MCH [27]. Vinholt PJ. et al. found a U-shaped association between mortality and platelet count [28]. Contrary to what this study found, a lesser platelet count during an ACS hospital stay is a reliable indicator of a better prognosis [29,30].

According to the ROC curve analysis, the most effective discriminative tools for predicting short-term mortality were MPV and RDW-SD. Numerous research studies have examined the predictive function of RDW in heart failure and ACS [31,32]. RDW had a respectable predictive capacity for all-cause death (C-statistics: 0.741, 95 % CI 0.694–0.788), according to ROC curves from a study by Talarico M et al. [33]. In fact, there is evidence linking platelet activity to the initiation and progression of atherosclerosis in several recent investigations, especially those involving patients with ACS [34]. Individuals with ACS have been found to have greater MPV [35], which is consistent with the results of this study. In the current investigation, MPV >10.7 was set as the maximum value for MPV's sensitivity and specificity. Similarly, MPV ≥10.5 fL was found to be predictive of plaque rupture in NSTE-ACS patients in a research by Wang J et al. [36].

RDW (AUC = 0.845) and MPV (AUC = 0.8014) had the best discriminative abilities to offer diagnostic and prognostic information when compared to the gold standard, highly sensitive troponin-I. According to Lippi G et al., MPV alone does not satisfy the standards for a successful patient assessment in ERs, but when combined with a highly sensitive troponin, it does merit additional study [37].

## 5 Strengths and limitations

This is the only study that we are aware of in Ethiopia that looks at the link between in-hospital mortality in patients with ACS and the dynamic profile of hematologic markers using repeated-measure analysis. One of the study's shortcomings is that patients were only watched while they were in the hospital. On the other hand, we think that a longer follow-up period for these patients could provide more insight into the predictive value of hematologic markers for the overall mortality rate of patients with ACS.

## 6 Conclusion

Ultimately, this study demonstrated that hematological markers, similar to other risk scores like GRACE and HEART risk stratification techniques, might be utilized as a rapid and effective means to evaluate patients' mortality risk if they had ACS. RDW-SD, MCV, MPV, and platelet counts are independent indicators of hospital mortality that indicate the basic pathophysiological mechanisms of diseases, namely systemic inflammation and hypoxemic impairment. According to the ROC curve analysis, the most effective discriminative tools for predicting short-term mortality were MPV and RDW-SD. Hematological indices are a widely available, reasonably priced, and crucial tool for intrahospital clinical surveillance when utilized by the multidisciplinary hospital team in their daily work. This is the main benefit of employing them to forecast the prognosis of patients with ACS.

## Data availability

Upon request, the corresponding author will provide the data supporting the study's conclusions. The information in the data could jeopardize research participants' privacy, hence they are not publicly accessible.

## CRediT authorship contribution statement

**Samuel Tadesse:** Writing – review & editing, Writing – original draft, Supervision, Software, Resources, Project administration, Methodology, Investigation, Formal analysis, Data curation, Conceptualization. **Elsah Tegene:** Writing – review & editing, Supervision, Resources, Conceptualization. **Daniel Yilma:** Writing – review & editing, Supervision, Methodology, Investigation, Formal analysis, Conceptualization. **Tilahun Yemane:** Writing – review & editing, Resources, Methodology, Formal analysis, Data curation, Conceptualization. **Esayas Kebede Gudina:** Writing – review & editing, Resources, Project administration, Methodology, Investigation, Formal analysis, Data curation, Conceptualization. **Andualem Mossie:** Writing – review & editing, Supervision, Resources, Methodology, Investigation, Formal analysis, Data curation.

## Declaration of competing interest

The authors declare that they have no known competing financial interests or personal relationships that could have appeared to influence the work reported in this paper.
